# Relationship between Clinical Parameters and Brain Structure in Sporadic Amyotrophic Lateral Sclerosis Patients According to Onset Type: A Voxel-Based Morphometric Study

**DOI:** 10.1371/journal.pone.0168424

**Published:** 2017-01-17

**Authors:** Hee-Jin Kim, Mony de Leon, Xiuyuan Wang, Hyun Young Kim, Young-Jun Lee, Yeon-Ha Kim, Seung Hyun Kim

**Affiliations:** 1 Department of Neurology, College of Medicine, Hanyang University, Seoul, Korea; 2 Center for Brain Health, Department of Psychiatry, NYU School of Medicine, New York, New York, United States of America; 3 Department of Neurology, NYU School of Medicine, New York, New York, United States of America; 4 Department of Radiology, College of Medicine, Hanyang University, Seoul, Korea; 5 College of Nursing, Sungshin University, Seoul, Korea; Universitat Ulm, GERMANY

## Abstract

**Background and purpose:**

Amyotrophic lateral sclerosis (ALS) is a rapidly progressing, phenotypically heterogeneous neurodegenerative disease affecting mainly the motor neuron system. The present voxel-based morphometry (VBM) study investigated whether patterns of brain atrophy differ among sporadic ALS subtypes.

**Material and methods:**

Sporadic ALS patients (n = 62) with normal cognition and age-matched healthy controls (n = 57) were included in the study. ALS patients were divided into limb- and bulbar-onset groups according to clinical manifestations at symptom onset (n = 48 and 14, respectively). Clinical measures were ALS Functional Rating Scale-Revised (ALSFRS-R) score, disease duration, and forced vital capacity (FVC). Patterns of brain atrophy between ALS subgroups were compared by VBM.

**Results:**

In limb-onset ALS patients, atrophy was largely confined to the motor cortex and adjacent pre- and postcentral regions. However, in the bulbar-onset group, affected regions were more widespread and included these same areas but also extended to the bilateral frontotemporal and left superior temporal and supramarginal gyri, and multiple regression analysis revealed that their ALSFRS-R scores were associated with extensive loss of gray matter while FVC was related to atrophy in subcortical regions of the left superior temporal gyrus. In limb-onset ALS patients, disease duration was related to the degree of atrophy in the motor and adjacent areas.

**Conclusion:**

Sporadic ALS subtypes show different patterns of brain atrophy. Neural networks related to limb and bulbar motor functions in each ALS subtype may underlie their distinct patterns of cerebral atrophy. That is, more extensive cortical and subcortical atrophy is correlated with greater ALSFRS-R severity and shorter disease duration in the bulbar-onset subtype and may explain the poor prognosis of these patients.

## Introduction

Amyotrophic lateral sclerosis (ALS) is a fatal and rapidly progressing neurodegenerative disease affecting both upper and lower motor neuron systems [1]. Despite the elucidation of genetic and molecular mechanisms of motor neuron cell death in ALS, the selectivity of affected motor neuron areas is not well understood and there are no effective therapeutic agents available for treatment [2]. The recent identification of novel genes linking ALS with frontotemproral dementia and the emerging concept of multisystem proteinopathies have changed the widely held belief that ALS affects only the motor neuron system [3, 4]. In addition, ALS patients manifesting only motor symptoms typically show heterogeneous clinical progression, and their prognosis can be predicted by the initial clinical presentation of either limb or bulbar onset, a reduced score on the revised Amyotrophic Lateral Sclerosis Functional Rating Scale (ALSFRS-R), and decreased forced vital capacity (FVC) [5].

Our recent data has demonstrated that diverse frontal dysfunction occurs in about 50% of sporadic ALS patients who harbor no known mutations in causative genes such as *C9ORF72*, *SOD1*, *FUS*, *and TDP43* [3, 6]. We also found that cognitive impairment was negatively correlated with patient prognosis and survival [7]. Based on these findings, the present study investigated whether ALS subtypes with normal cognition but with distinct clinical presentation show differences in terms of changes in brain structure. We evaluated differences in regional brain volumes between limb- and bulbar-onset ALS patients to test the hypothesis that different patterns of brain atrophy are associated with clinical measures including ALSFRS-R score, disease duration, and FVC.

## Materials and Methods

### Subjects

A total of 319 sporadic ALS patients were recruited between 2008 and 2012 for this study who met the criteria for clinically definite or probable (laboratory-supported) ALS [8]; of these, 96 were excluded due to systemic medical disease (n = 3), psychiatric illness or use of psychiatric drugs (n = 9), other neurological disease (e.g., stroke, trauma, or learning disability) (n = 16), or insufficient neuropsychological testing (n = 68). Based on neuropsychological test results and magnetic resonance imaging (MRI) data, 103 ALS patients who showed abnormal cognitive findings based on the Diagnostic and Statistical Manual of Mental Disorders, Fourth Edition criteria [9] were evaluated on most tasks in the Seoul Neuropsychological Screening Battery [7, 10]. An additional 58 patients from the remaining 120 who were cognitively normal [7] were also excluded based on the following criteria: age over 75 years; simultaneous presentation of limb motor signs and bulbar symptoms; and history of respiratory support (tracheostomy, non-invasive ventilation, or oxygen inhalation). Ultimately, 62 sporadic ALS patients with limb (n = 48) or bulbar (n = 14) onset with analyzable MRI data were included in the study (S1 Fig).

We analyzed clinical features of the disease. Onset symptoms were collected by experienced medical staff through interviews with patients or their family members. ALS subgroups were classified based on initial clinical presentation, as follows: limb onset cases were defined as having limb motor symptoms or signs (weakness or atrophy or fasciculation) and no bulbar signs or symptoms at the time point of initial manifestations. Bulbar onset cases were defined as having only bulbar signs or symptoms including dysarthria, swallowing difficulty and tongue fasciculation and no limb weakness.

The control group consisted of 57 sex- and age-matched healthy subjects. We obtained written informed consent to participate and enrollment of cohort. The ethics committees/IRBs approved this consent procedure. The study protocol was approved by the Institutional Review Board of Hanyang University Hospital, Seoul, South Korea (HYU 2010-04-011).

### Clinical measurements

ALSFRS-R is used to evaluate the functional status of ALS patients [11] based on 12 items, including: bulbar symptoms, limb/axial muscle function, and respiratory symptoms, each of which are rated on a scale of 0–4. Total functional disability scores range from 0 (maximum disability) to 48 (normal) points. Patients’ respiratory function was estimated by FVC (% of predicted normal value) using a conventional pulmonary function test [12]. Disease duration was defined as the time interval from initial symptom onset to the time of MRI, at which time other clinical parameters were also measured.

### Image acquisition

MRI was carried out using a 3.0-T Achieva system (Phillips, Best, The Netherlands) equipped with a standard quadrature head coil. Structural MRI sequences were obtained as a volumetric three-dimensional spoiled fast gradient echo with the following parameters: repetition time = 7.3 ms; echo time = 2.7 ms; slice thickness = 1.0 mm; flip angle = 13°; and field of view = 256 × 256 mm. The volume consisted of 200 contiguous coronal sections covering the entire brain.

### VBM analysis

VBM is a fully automated, whole-brain technique that enables measurement of regional brain volumes based on voxel-wise comparison of gray and white matter volumes using Statistical Parametric Mapping 12 software (SPM12; Functional Imaging Laboratory, Wellcome Department of Imaging Neuroscience, Institute of Neurology, London, UK; http://www.fil.ion.ucl.ac.uk/spm) running on Matlab 2015b (Matworks, Natick, MA, USA). Structural images were bias-corrected, tissue-classified, and normalized to a standard template using high-dimensional DARTEL normalization. Images were segmented into gray, white, and cerebrospinal fluid compartments using VBM8 preprocessing in SPM12. The ‘check data quality’ function was used to verify segmentation results. Gray and white matter volumes were smoothed using a 12-mm full width at half-maximum Gaussian filter.

### Statistical analysis

Differences in demographic factors and clinical parameters of each ALS subtype were evaluated with the t test for independent samples for continuous variables and with the χ^2^ test for categorical variables with Bonferroni correction in the post-hoc analysis. Data are presented as median values with standard deviation and ranges. The normality of data distributions was tested with the Kolmogorov–Smirnov test. P values < 0.05 were considered statistically significant. Statistical analyses were performed using SPSS v.22 software (SPSS Inc., Chicago, IL. USA). MR data were analyzed with the two-sample t test to compare regional volumes between ALS patients and controls. Group differences between bulbar- and limb-onset ALS were assessed using SPM 12. Age, sex, and total intracranial volume were corrected for as confounding variables in an analysis of covariance. Specifically, single linear regression analyses with clinical parameters were performed in limb- and bulbar-onset ALS for the following contrasts: ALSFRS-R composite score, disease duration, and FVC (%). We accepted a statistical threshold of P < 0.001 (uncorrected) with an extent threshold of 100 voxels and P < 0.05 (corrected) using family-wise error methods [13].

## Results

### Clinical characteristics of the study population

Limb- and bulbar-onset ALS groups did not differ in terms of demographic factors including sex and age or clinical parameters including ALSFRS-R score and FVC. Disease progression—which was estimated from the decline in ALSFRS-R score from the time of symptom onset determined by MRI (48-ALSFRS-R at MRI/disease duration in months)—showed more rapid progression in the bulbar-onset subtype, although the difference was not significant. However, disease duration was significantly shorter in this group as compared to limb-onset ALS patients (15.64 ± 8.12 vs. 26.17 ± 19.12, P < 0.05) (Table 1).

**Table 1:** Demographic and clinical characteristics of ALS patients and control subjects.

	ALS	Limb-onset ALS	Bulbar-onset ALS	Control
Characteristics				
Number of subjects with probable or definite ALS	62 (56/6)	48 (46/2)	14 (10/4)	57
Sex (male/female)*	33/29	29/19	4/10	20/23
Age (years)*	52.73 ± 10.05	50.95 ± 11.10	56.78 ± 7.16	51.09 ± 5.17
ALSFRS-R score*	37.31 ± 6.46	37.23 ± 6.08	37.50 ± 7.44	N/A
Months from ALS disease onset*	28.07 ± 18.99	26.17 ± 19.12^†^	15.64 ± 8.12^†^	N/A
Disease progression*^‡^	0.58 ± 0.45	0.53 ± 0.38	0.74 ± 0.64	N/A
FVC*	61.27 ± 25.03	64.35 ± 25.22	52.78 ± 23.64	N/A

* Values represent mean sent.

^†^P = 0.05 (=^2^ test with post hoc Bonferroni correction).

^‡^ Progression rate was estimated from the decline in ALSFRS-R score after symptom onset (48-ALSFRS-R at MRI/disease duration).

ALS, amyotrophic lateral sclerosis; ALSFRS-R, Korean version of ALS Functional Rating Scale—Revised; FVC, forced vital capacity.

### Patterns of brain atrophy in gray and white matter

We compared regions exhibiting gray and white matter atrophy in ALS patients and controls. In the former, we observed reduced gray matter density in the bilateral frontal cortex (especially the middle frontal gyrus and right precentral and postcentral gyrus), cerebellum, left superior temporal gyrus, left inferior parietal gyrus, and insula (S1 Table and S2A Fig). Analysis of white matter revealed that the right paracentral lobule, right insula, right Rolandic operculum, and right superior temporal, left postcentral, and left superior frontal gyri were markedly atrophied in ALS patients relative to controls (S1 Table and S2B Fig).

### Differences in brain atrophy between and within limb- and bulbar-onset ALS subtypes

**Fig 1:**
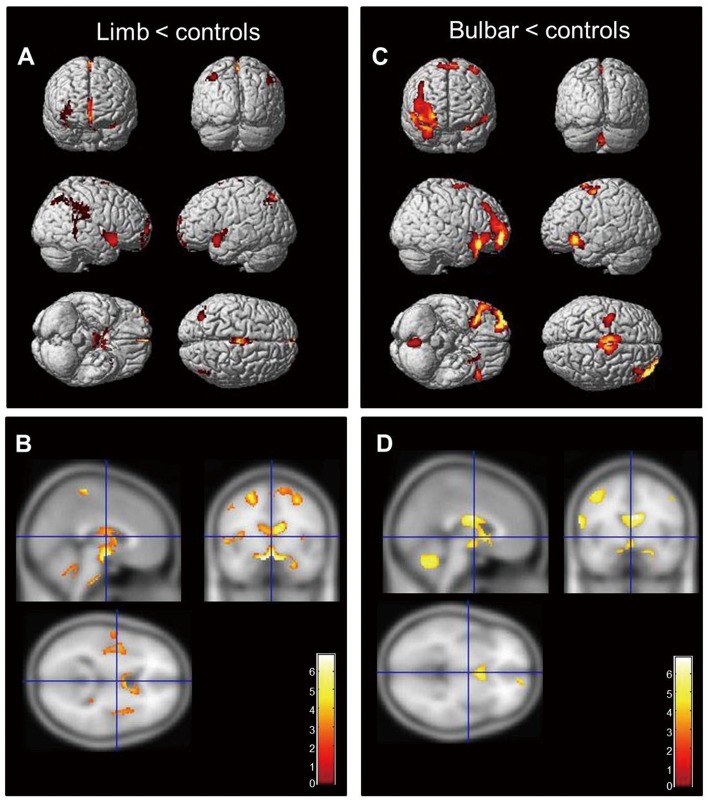
Regional atrophy in ALS patients relative to controls. (A, C) Limb-onset ALS showing gray matter atrophy in bilateral inferior frontal, left superior frontal, and left precentral gyri, SMA, and thalamus (A). In bulbar-onset ALS, atrophy was detected in bilateral frontal lobes, left superior temporal pole, and left inferior parietal, precentral, and postcentral gyri relative to healthy controls (C). In limb-onset ALS, white matter atrophy occurred around motor and association areas (SMA and around central lobules). (B, D) In the bulbar type, multiple areas of the white matter tract in extra-motor areas (SMA and inferior and superior frontal gyri) as well as the motor area were affected (displayed at P < 0.001, uncorrected, extended threshold = 100 voxels). The colored bar represents the T score.

We separately compared atrophy patterns in the limb- and bulbar-onset ALS subtypes to that of controls. Limb-onset ALS patients showed more gray matter atrophy in bilateral supplementary motor areas (SMA) and inferior frontal and superior temporal areas relative to controls (Fig 1A and S2 Table), whereas the bulbar-onset subtype showed SMA and inferior frontal area atrophy, but exhibited more extensive atrophy than the limb-onset group (Fig 1C and S2 Table).

**Fig 2:**
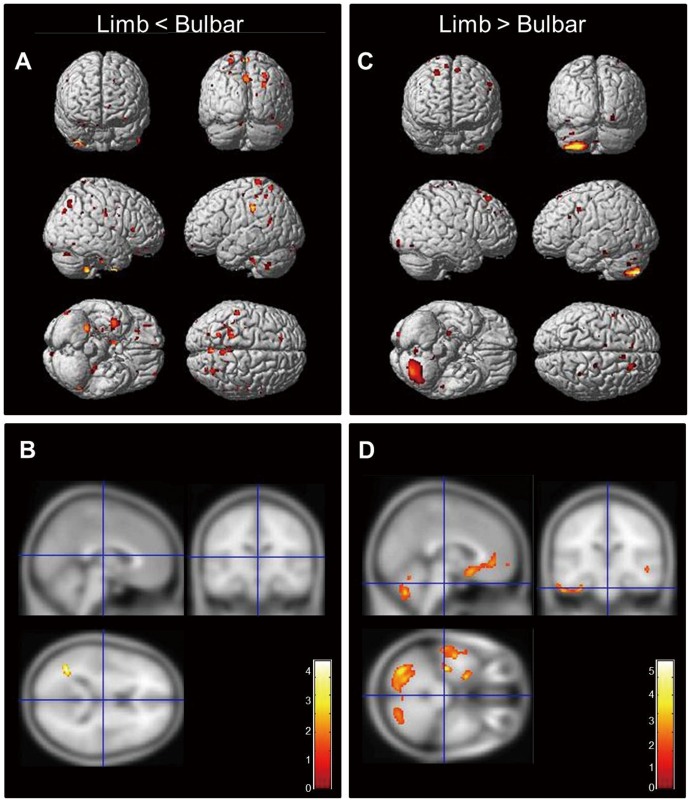
Comparative analysis of regional gray and white matter atrophy between limb- and bulbar-onset ALS patients. (A, B) The limb-onset subtype was characterized by decreased gray matter volume around motor areas (pre- and post-central and medial superior frontal areas) relative to the bulbar-onset subtype. (C, D) The bulbar-onset subtype exhibited atrophy in both gray and white matter in extra-motor areas—i.e., bilateral frontal, temporal, and cerebellar areas as compared to the limb type (displayed at P < 0.001, uncorrected, extended threshold = 100 voxels). The colored bar represents the T score.

Compared to controls, limb-onset ALS patients showed white matter atrophy in motor and adjacent areas. In contrast, the bulbar-onset group showed more widespread involvement of white matter tracts, including motor and extra-motor regions (Fig 1B, 1D and S3 Table). Atrophic changes in the bulbar onset subtype were more widespread than in limb-onset patients, extending to extra-motor regions including SMA, superior frontal, inferior temporal, and cerebellar areas (Fig 2A and 2C). Similarly, white matter atrophy was limited to the left middle temporal regions in limb-onset ALS, whereas bulbar-onset patients showed multiple affected areas in the frontotemporal region and bilateral cerebellum (Fig 2B and 2D). Comparative data on the patterns of brain atrophy in each ALS subtype are summarized in Table 2.

**Table 2:** Summary of atrophied regions in limb- vs. bulbar-onset ALS.

	Atrophied areas*	Limb-onset ALS	Bulbar-onset ALS
Gray matter	Compared controls	SMA (B)	SMA (B)
		Medial orbitofrontal (L)	Precentral (L)
		Medial superior frontal (L)	Superior frontal (L)
		Inferior parietal (L)	Inferior frontal, orbitalis (B)
		Superior temporal pole (L)	Thalamus (B)
		Insular (L)	
	Comparison within ALS subgroups	Precentral (B)	SMA (R)
		Postcentral (B)	Superior frontal (B)
		Medial superior frontal (B)	Inferior temporal (L)
			Cerebellum (B)
White matter	Compared controls	SMA (L)	SMA (B)
		Precentral (R)	Precentral (R)
		Paracentral lobule (L)	Postcentral (L)
		Superior temporal (L)	Superior frontal (R)
		Heschl’s gyrus (L)	Middle frontal (B)
		Insula (B)	Medial superior frontal (R)
		Cerebellum vermis	Cerebellum (L)
	Comparison within ALS subgroups	Middle temporal (L)	Superior frontal (B)
			Middle temporal (R)
			Inferior temporal (L)
			Cerebellum (B)

*All atrophied areas were based on AAL regions.

ALS, amyotrophic lateral sclerosis; B, bilateral; L, left; R, right; SMA, supplementary motor area.

### Relationship between clinical parameters and atrophic patterns in limb- vs. bulbar-onset ALS

**Fig 3:**
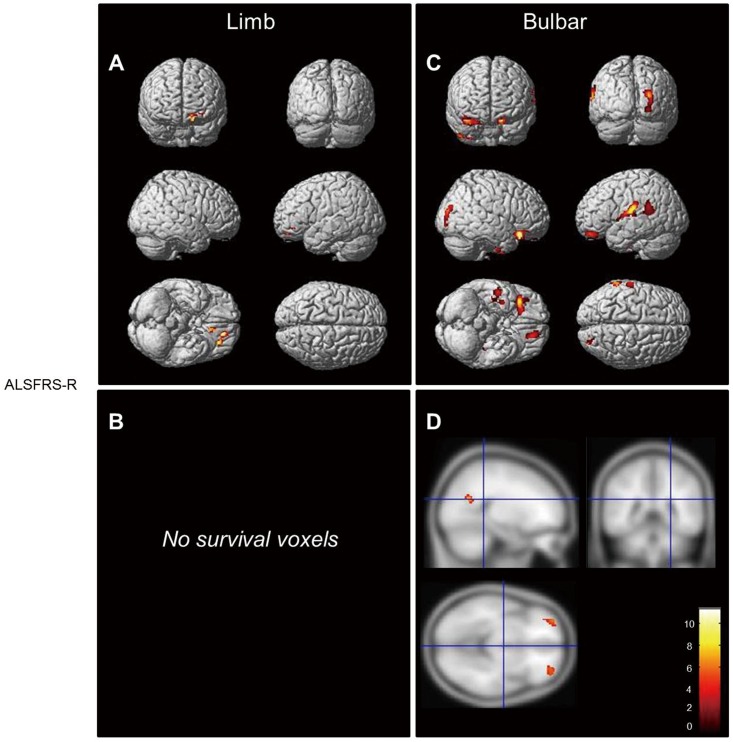
Different atrophic patterns in bulbar- and limb-onset ALS patients according to ALSFRS-R scores. (A, B) The bulbar-onset group showed atrophy in the bilateral frontal, left superior temporal, and supramarginal gyri (A). The limb-onset group showed atrophy in the left orbitofrontal area (B) (displayed at P < 0.001, uncorrected, extended threshold 100 voxels). The colored bar represents the T score.

ALSFRS-R score was associated with atrophy in multifocal gray matter areas including the bilateral frontal, left superior, and supramarginal gyri in bulbar-onset patients. However, limb-onset patients showed atrophy in the left orbitofrontal area (Fig 3A and 3C); in this group but not the bulbar-onset group, atrophy in the motor and left frontal areas was related to disease duration (S2C and S2D Fig). In the latter patients, focal atrophy in the subcortex of the left superior temporal gyrus was associated with FVC (S2E and S2F Fig) whereas in the former, FVC was unrelated to changes in gray and white matter volume.

## Discussion

Recent evidence suggests that ALS is a multisystem neurodegenerative disease involving both sensory and extrapyramidal systems [14]. Over 50% of ALS cases show cognitive impairment involving many components of the central nervous system [15, 16]. Neuroimaging studies can provide insight into the relationship between anatomical and functional abnormalities in motor and neural networks that depend on afferent and efferent connections [14]. Our imaging study evaluating changes in gray and white matter in sporadic ALS patients with normal cognitive function and only pure motor manifestations can clarify the clinical correlates. To this end, the current study examined differences in atrophy between limb- and bulbar-onset ALS and the associated clinical changes. Widespread cortical and subcortical atrophy in the motor and adjacent regions as well as extra-motor regions were observed in ALS patients. Furthermore, the bulbar-onset subtype showed multifocal and more extensive atrophy in extra-motor gray and white matter than limb-type ALS. These findings suggest that atrophy occurs at the initial anatomical site linked to progressive motor deterioration.

We found the ALSFRS-R score was highly correlated with the severity of gray and white matter atrophy in bulbar-onset ALS. Others have reported that clinical progression in this subtype is more rapid than in limb-onset ALS based on ALSFRS-R score [17]. We observed a similar trend; this may be explained by more extensive atrophy in multifocal, extra-motor gray matter areas, including bilateral frontal lobes and the left supramarginal gyrus as well as motor regions. In contrast, in limb-onset ALS patients, only left orbitofrontal atrophy was associated with ALSFRS-R. These results suggest that the patterns and extent of brain atrophy in our study may be useful for predicting the progression of ALS.

Motor-related regional atrophy (e.g., in the SMA) was found to be independent of onset type. We also found that bulbar-onset patients exhibited atrophy in the bilateral frontotemporal lobes irrespective of disease duration. In contrast, in the limb-onset subtype, atrophy was confined to the precentral cortex and adjacent regions—that is, to anatomical regions related to motor control [5]. In bulbar-onset patients, more extensive anatomical changes may precede bulbar manifestations even at an early stage. Our findings may be related to prior observations of poor prognosis in this ALS subtype [18–22]. Interestingly, despite the shorter disease duration in these patients, gray and white matter atrophy was more widespread than in those with limb-onset ALS corresponding to their poor prognosis [21], [22]. One report described the importance of the corpus callosum in bulbar ALS as a predictor of poor prognosis [23]; however, we found no evidence of corpus callosum involvement in this study, which may due to the heterogeneity of ALS patients [24] and different selection criteria that were used for patient recruitment—for instance, the inclusion of only cognitively normal ALS patients in the present study.

FVC is an another useful parameter for predicting ALS patient prognosis [19, 20]. In contrast to a previous report [25], there was no evidence of a relationship between respiratory function and brain atrophy, except for the observation that the left superior temporal subcortex is involved in bulbar-onset ALS. The mean FVC of patients enrolled in this study was more than 60% of the predicted normal value and most patients did not present severe respiratory insufficiency; moreover, there was no difference in FVC between the two ALS subtypes, which may be explain why respiratory symptoms were not correlated with brain atrophy. Another plausible explanation is that the link between respiratory function and ALS prognosis is related to weakness of respiratory muscles rather than to cortical or subcortical changes.

A previous study reported cortical thinning around primary motor areas in patients with spinal- but not bulbar-onset ALS as compared to healthy controls [26]. Another group found that the site of disease onset (bulbar/lower limb) showed the most pronounced thinning in the corresponding area of the motor cortex, specifically in the region harboring upper motor neurons [27]. Although brain volume differed between limb- and bulbar-onset subtypes of ALS, they showed similar total gray or white matter volumes [28]. ALS patients exhibit localized bilateral deficits in gray matter volume centered in Brodmann’s areas 8, 9, and 10 along with bilateral reductions in white matter volume from the precentral gyrus to the internal capsule and brainstem relative to healthy control subjects, consistent with the course of the corticospinal tract. In contrast, there was no loss in gray matter volume in the precentral gyrus [28]. VBM is relatively sensitive to extra-motor—especially frontotemporal cerebral—changes [29]. Although multimodal MRI studies of ALS patients can be used to characterize disease-specific pathology, they are limited by inconsistent results due to small sample size, suboptimal patient characterization, lack of post-mortem validation of imaging findings, heterogeneity of disease progression, and a paucity of longitudinal data [30, 31]. Previous VBM analyses of ALS have yielded inconsistent findings [14], possibly due to diverse factors such as covariates [32], matching, sampling severity, sample size [33], template normalization [34], threshold masking [35], imaging protocols [36], and statistical modeling [37]. Although volumetric imaging and similar approaches cannot recapitulate neuropathological findings of the central nervous system, the recent development of image acquisition and analytical tools has provided new insights into neural networks involved in ALS [14] as well as evidence of extra-motor involvement [14, 38–42]. According to recent meta-analysis data based on analysis of 116 studies reported that the MRI biomarkers appear to be well correlated with disease severity, duration and progression and are more sensitive not only in the spinal cord motor regions but also more extensive brain regions [43]. This study suggest that VBM could be one of reliable image biomarkers to predict prognosis and reveal clinically silent involvement of ALS, even though there are some limitations such as the clinical various phenotypes and the lack of large and longitudinal studies [43].

This study had some limitations. Firstly, there was a large discrepancy between the two clinical groups even though it was a prospective cohort study. Secondly, observations on white matter lesions must be interpreted with caution. Diffusion tensor imaging (DTI) is a more useful tool than VBM for detecting network degeneration in ALS and evaluating network integrity than selected measurements of grey or white matter [30, 43–45]. There is a need for large cohorts with long term DTI and clinical follow-up for solving this issue. Thirdly, we attempted to obtain more reliable results for the FWE correction. However, we failed to obtain positive results under FWE correction, even though there were positive finds in the uncorrected analyses (p<0.001). Studies with larger sample size will typically be able to apply the harsh FWE correction for multiple comparisons, however, it may be difficult to obtain positive findings in a study with a small sample size [13, 46]. Many other researchers have reported image results of ALS using an uncorrected p value [46, 47]. There is possibility of over-interpretation in potentially false positives observed significant uncorrected results in this study.

In summary, this study found that sporadic ALS patients with normal levels of cognition and pure motor symptoms show multiple sites of cortical and subcortical atrophy in areas that extend beyond motor regions. This was especially apparent in bulbar-onset patients, suggesting that neural network dysfunction—particularly salient and central executive networks such as insular and fronto-orbital cortical and associated subcortical regions—underlies ALS, with the different subtypes showing distinct atrophic patterns. Given that ALS involves focal degeneration that spreads to through upper and lower motor levels [48], it is assumed that bulbar-onset patients experience more rapid and contiguous spreading of brain atrophy. Therefore, VBM analysis of gray and white matter atrophy in ALS can provide a basis for predicting ALS progression and prognosis.

## Supporting Information

S1 Fig Selection of study participants. A total of 319 clinically probable laboratory-supported ALS patients were enrolled in the study. Neuropsychological testing was carried out on 223 patients; 120 showed normal cognitive function, and 62 underwent neuroimaging for VBM analysis. The control group consisted of 57 age-matched subjects.
(TIF)

S2 Fig (A, B) Patterns of atrophy in ALS patients relative to healthy controls. (C, D) Atrophied regions included motor and extramotor areas of gray and white matter. Different patterns were observed in bulbar- and limb-onset ALS patients according to disease duration. Patients with limb-onset disease showed atrophy in the left frontal and motor subcortical areas within the primary motor region. (E, F) Decreased FVC was correlated with atrophy in bilateral superior temporal and orbitofrontal subcortical areas in the bulbar-onset subtype (displayed at P < 0.001, uncorrected, extended threshold = 100 voxels). The colored bar represents the T score.
(TIF)

S1 Table Relative decrease in brain volume in all ALS patients compared to controls. There was reduced gray matter density in the bilateral frontal cortex, cerebellum, left superior temporal gyrus, left inferior parietal gyrus, and insula in ALS.
(DOCX)

S2 Table Relative decrease in gray matter volume of bulbar and limb-onset ALS compared to controls. There were more gray matter atrophy in bilateral supplementary motor areas (SMA) and inferior frontal and superior temporal areas relative to controls in limb-onset ALS patients, whereas the bulbar-onset subtype showed SMA and inferior frontal area atrophy, but exhibited more extensive atrophy than the limb group.
(DOCX)

S3 Table Relative decrease in white matter volume of bulbar- and limb-onset ALS patients compared to controls. Limb-onset ALS patients showed white matter atrophy in motor and adjacent areas. In contrast, the bulbar-onset group showed more widespread involvement of white matter tracts, including motor and extra-motor regions.
(DOCX)
