# Supplementary figures and images for: Relationship between Clinical Parameters and Brain Structure in Sporadic Amyotrophic Lateral Sclerosis Patients According to Onset Type: A Voxel-Based Morphometric Study
Source: PLoS One. 2017 Jan 17;12(1):e0168424. doi: 10.1371/journal.pone.0168424 (PMC5240978; doi:10.1371/journal.pone.0168424)

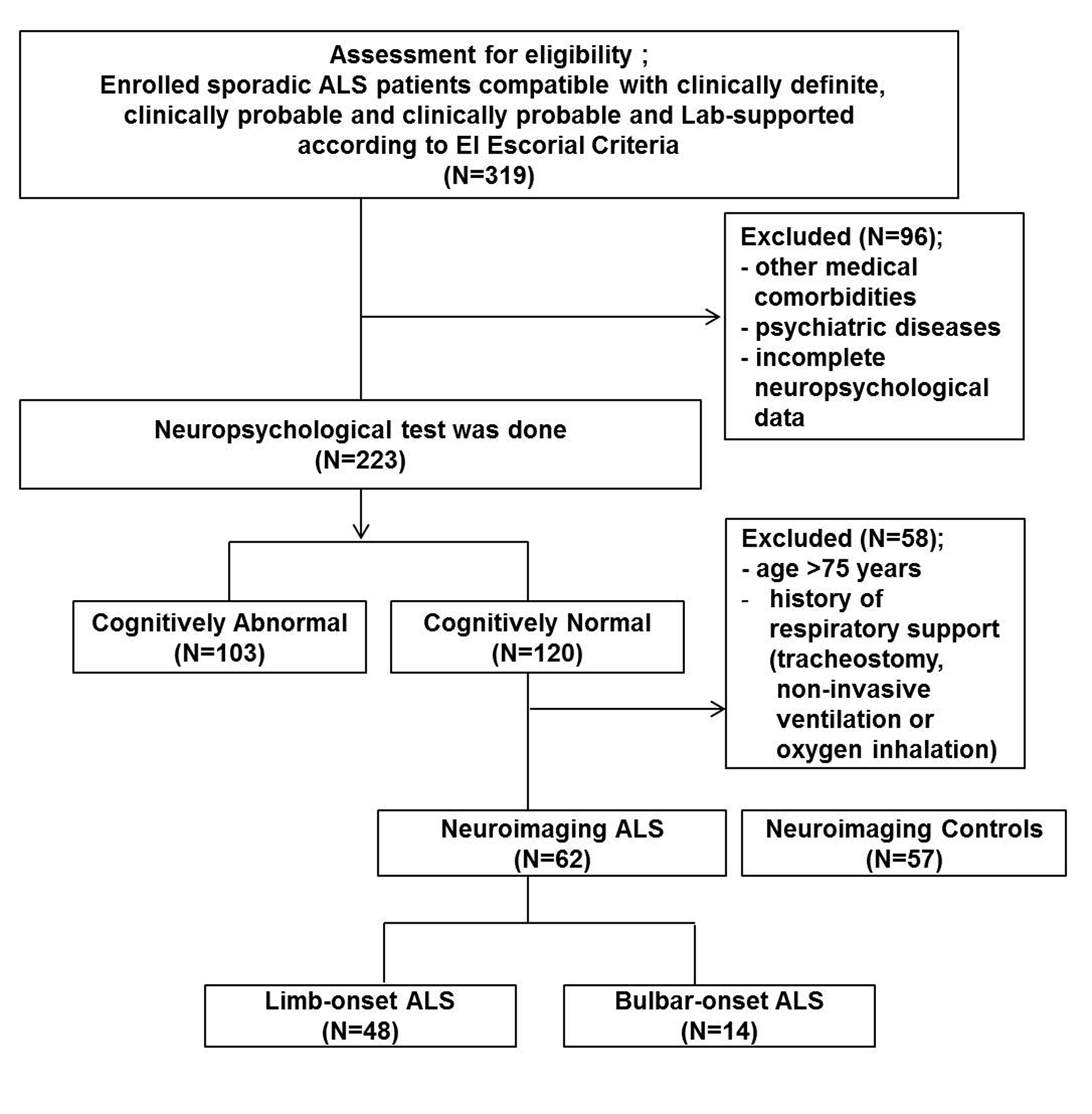

Supplement: S1 Fig — A total of 319 clinically probable laboratory-supported ALS patients were enrolled in the study. Neuropsychological testing was carried out on 223 patients; 120 showed normal cognitive function, and 62 underwent neuroimaging for VBM analysis. The control group consisted of 57 age-matched subjects. (TIF) [file pone.0168424.s001.tif]

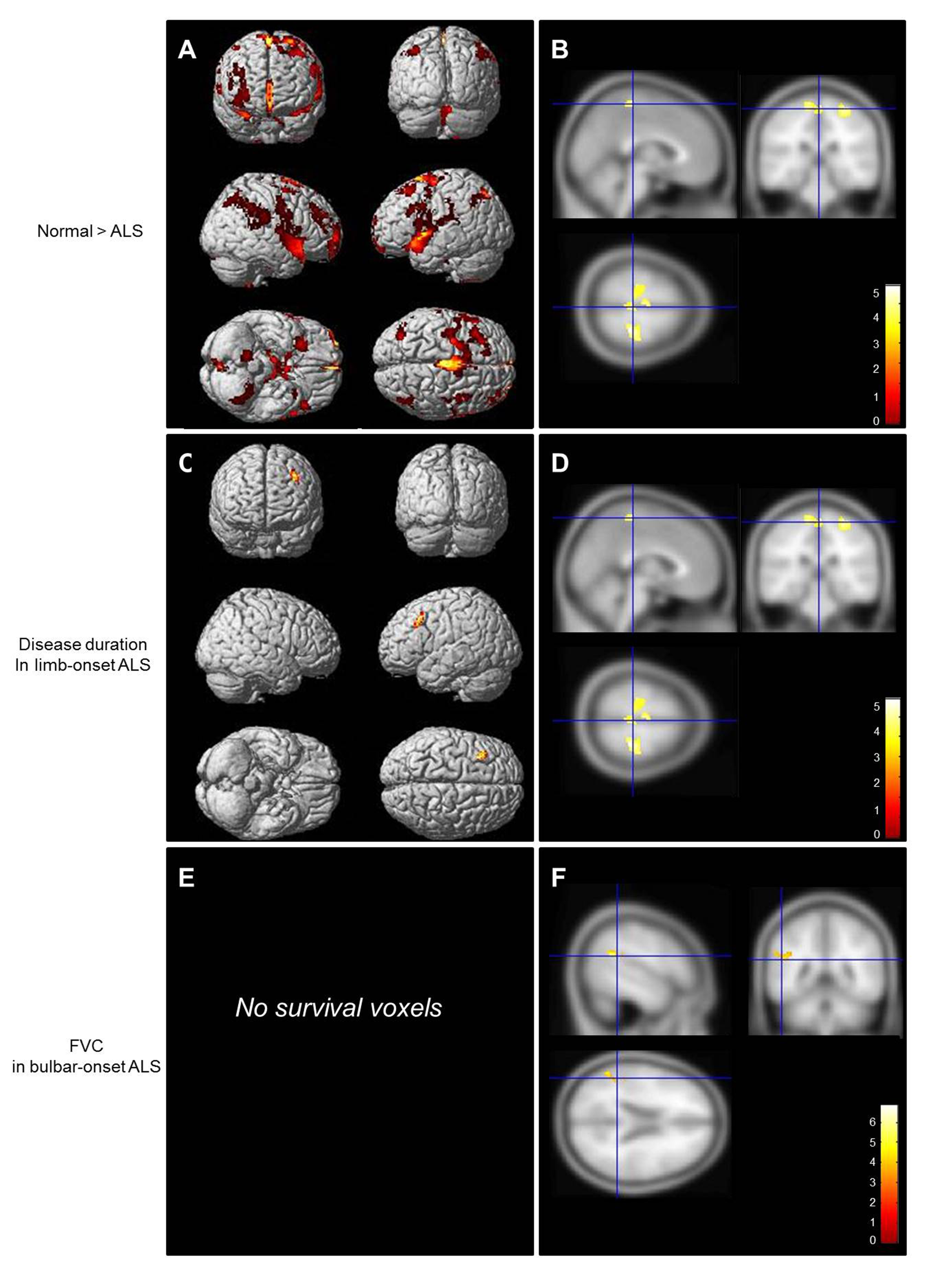

Supplement: S2 Fig — (A, B) Patterns of atrophy in ALS patients relative to healthy controls. (C, D) Atrophied regions included motor and extramotor areas of gray and white matter. Different patterns were observed in bulbar- and limb-onset ALS patients according to disease duration. Patients with limb-onset disease showed atrophy in the left frontal and motor subcortical areas within the primary motor region. (E, F) Decreased FVC was correlated with atrophy in bilateral superior temporal and orbitofrontal subcortical areas in the bulbar-onset subtype (displayed at P < 0.001, uncorrected, extended threshold = 100 voxels). The colored bar represents the T score. (TIF) [file pone.0168424.s002.tif]
