# Supplementary material for: Relationship between Clinical Parameters and Brain Structure in Sporadic Amyotrophic Lateral Sclerosis Patients According to Onset Type: A Voxel-Based Morphometric Study
Source: PLoS One. 2017 Jan 17;12(1):e0168424. doi: 10.1371/journal.pone.0168424 (PMC5240978; doi:10.1371/journal.pone.0168424)
Supplement: S1 Table — There was reduced gray matter density in the bilateral frontal cortex, cerebellum, left superior temporal gyrus, left inferior parietal gyrus, and insula in ALS. (DOCX) [file pone.0168424.s003.docx]

| \| Supporting 1 Table. Relative decrease in brain volume in all ALS patients compared to controls \| \| \| \| \| \| \| \| \| --- \| --- \| --- \| --- \| --- \| --- \| --- \| --- \| \|  \|  \|  \| MNI-space \| \| \|  \|  \| \| AAL regions \| Side \| Clusters \| x \| y \| z \| T-value \| P-value \| \| **Gray matter** \|  \|  \|  \|  \|  \|  \|  \| \| Superior temporal pole \| Left \| 3481 \| -36 \| 12 \| -18 \| 5.20 \| 0.000 \| \| Middle frontal gyrus \| Left \| 3481 \| -40 \| 22 \| 56 \| 5.20 \| 0.000 \| \| Postcentral gyrus \| Right \| 765 \| 66 \| -2 \| 38 \| 4.53 \| 0.001 \| \| Superior frontal gyrus \| Right \| 561 \| 32 \| 66 \| 4 \| 4.83 \| 0.005 \| \| Middle frontal gyrus \| Right \| 561 \| 36 \| 54 \| 30 \| 4.21 \| 0.005 \| \| Medial orbitofrontal cortex \| Left \| 590 \| 0 \| 60 \| 0 \| 4.67 \| 0.004 \| \| Medial frontal gyrus (superior) \| Left \| 590 \| 0 \| 68 \| 14 \| 4.26 \| 0.004 \| \| [Lobule IV, V of cerebellar hemisphere](http://neuro.imm.dtu.dk/w/index.php?title=Category:Left_lobule_IV,_V_of_cerebellar_hemisphere&action=edit&redlink=1) \| Left \| 202 \| -12 \| -34 \| -14 \| 4.20 \| 0.068 \| \| [Crus II of cerebellar hemisphere](http://neuro.imm.dtu.dk/w/index.php?title=Category:Right_crus_II_of_cerebellar_hemisphere&action=edit&redlink=1) \| Right \| 215 \| 4 \| -84 \| -24 \| 4.04 \| 0.060 \| \| Inferior parietal lobule \| Left \| 179 \| -36 \| -72 \| 52 \| 5.39 \| 0.083 \| \| Middle frontal gyrus \| Right \| 137 \| 46 \| 0 \| 62 \| 4.43 \| 0.125 \| \| Middle frontal gyrus \| Right \| 137 \| 42 \| 8 \| 62 \| 4.33 \| 0.125 \| \| Precentral gyrus \| Right \| 137 \| 46 \| -18 \| 68 \| 3.58 \| 0.125 \| \|  \|  \|  \|  \|  \|  \|  \|  \| \| **White matter** \|  \|  \|  \|  \|  \|  \|  \| \| Paracentral lobule \| Right \| 8225 \| 6 \| -28 \| 74 \| 6.86 \| 0.000 \| \| Postcentral gyrus \| Left \| 8225 \| -24 \| -28 \| 64 \| 5.85 \| 0.000 \| \| Superior frontal gyrus \| Left \| 8225 \| -22 \| 2 \| 64 \| 5.56 \| 0.000 \| \| Insular \| Right \| 348 \| 40 \| -2 \| 8 \| 4.40 \| 0.012 \| \| Insular \| Right \| 348 \| 42 \| -10 \| 4 \| 4.00 \| 0.012 \| \| Superior temporal gyrus \| Right \| 114 \| 62 \| -8 \| 8 \| 4.56 \| 0.124 \| \| Rolandic operculum \| Right \| 114 \| 62 \| 4 \| 14 \| 3.82 \| 0.124 \| \|  \|  \|  \|  \|  \|  \|  \|  \| |
| --- | --- | --- | --- | --- | --- | --- | --- | --- | --- | --- | --- | --- | --- | --- | --- | --- | --- | --- | --- | --- | --- | --- | --- | --- | --- | --- | --- | --- | --- | --- | --- | --- | --- | --- | --- | --- | --- | --- | --- | --- | --- | --- | --- | --- | --- | --- | --- | --- | --- | --- | --- | --- | --- | --- | --- | --- | --- | --- | --- | --- | --- | --- | --- | --- | --- | --- | --- | --- | --- | --- | --- | --- | --- | --- | --- | --- | --- | --- | --- | --- | --- | --- | --- | --- | --- | --- | --- | --- | --- | --- | --- | --- | --- | --- | --- | --- | --- | --- | --- | --- | --- | --- | --- | --- | --- | --- | --- | --- | --- | --- | --- | --- | --- | --- | --- | --- | --- | --- | --- | --- | --- | --- | --- | --- | --- | --- | --- | --- | --- | --- | --- | --- | --- | --- | --- | --- | --- | --- | --- | --- | --- | --- | --- | --- | --- | --- | --- | --- | --- | --- | --- | --- | --- | --- | --- | --- | --- | --- | --- | --- | --- | --- | --- | --- | --- | --- | --- | --- | --- | --- | --- | --- | --- | --- | --- | --- | --- | --- | --- | --- | --- | --- | --- | --- | --- | --- | --- | --- | --- | --- | --- | --- | --- | --- | --- | --- | --- | --- | --- | --- | --- | --- | --- | --- | --- | --- | --- | --- | --- | --- | --- | --- | --- | --- | --- | --- |
