# Supplementary material for: Relationship between Clinical Parameters and Brain Structure in Sporadic Amyotrophic Lateral Sclerosis Patients According to Onset Type: A Voxel-Based Morphometric Study
Source: PLoS One. 2017 Jan 17;12(1):e0168424. doi: 10.1371/journal.pone.0168424 (PMC5240978; doi:10.1371/journal.pone.0168424)
Supplement: S2 Table — There were more gray matter atrophy in bilateral supplementary motor areas (SMA) and inferior frontal and superior temporal areas relative to controls in limb-onset ALS patients, whereas the bulbar-onset subtype showed SMA and inferior frontal area atrophy, but exhibited more extensive atrophy than the limb group. (DOCX) [file pone.0168424.s004.docx]

| Supporting 2 Table. Relative decrease in gray matter volume of bulbar and limb-onset ALS compared to controls | | | | | | | | |
| --- | --- | --- | --- | --- | --- | --- | --- | --- |
|  |  |  |  | MNI-space | | |  |  |
| Type | AAL regions | Side | Clusters | x | y | z | T-value | P-value |
|  | |  |  |  |  |  |  |  |
| Limb | Supplementary motor area | Left | 402 | 0 | -12 | 64 | 4.19 | 0.007 |
|  | Supplementary motor area | Right | 402 | 2 | 4 | 72 | 3.53 | 0.007 |
|  | Medial orbitofrontal cortex | Left | 394 | 0 | 60 | 0 | 4.41 | 0.008 |
|  | Superior temporal pole | Left | 293 | -36 | 12 | -18 | 4.52 | 0.019 |
|  | Insula | Left | 293 | -44 | -2 | 0 | 3.65 | 0.019 |
|  | Inferior parietal lobule | Left | 126 | -36 | -72 | 52 | 5.27 | 0.104 |
|  | Medial frontal gyrus (superior) | Left | 394 | 0 | 68 | 16 | 4.14 | 0.104 |
|  |  |  |  |  |  |  |  |  |
| Bulbar | Inferior frontal gyrus (orbitalis) | Right | 3004 | 38 | 22 | -16 | 5.01 | 0.000 |
|  | Inferior frontal gyrus (orbitalis) | Right | 3004 | 20 | 56 | -24 | 4.85 | 0.000 |
|  | Supplement motor area | Left | 1072 | -6 | -6 | 62 | 4.70 | 0.000 |
|  | Supplement motor area | Left | 1072 | -6 | 4 | 70 | 4.49 | 0.000 |
|  | Supplement motor area | Right | 1072 | 8 | -8 | 68 | 4.51 | 0.000 |
|  | Inferior frontal gyrus (orbitalis) | Left | 729 | -42 | 20 | -10 | 4.71 | 0.002 |
|  | Thalamus | right | 195 | 8 | -14 | 18 | 3.99 | 0.080 |
|  | Precentral gyrus | Left | 231 | -38 | -4 | 62 | 4.18 | 0.059 |
|  | Superior frontal gyrus | Left | 231 | -26 | -8 | 60 | 3.87 | 0.059 |
|  | Thalamus | Left | 174 | -12 | -22 | 20 | 3.98 | 0.096 |
|  |  |  |  |  |  |  |  |  |
|  |  |  |  |  |  |  |  |  |
