# Supplementary material for: Relationship between Clinical Parameters and Brain Structure in Sporadic Amyotrophic Lateral Sclerosis Patients According to Onset Type: A Voxel-Based Morphometric Study
Source: PLoS One. 2017 Jan 17;12(1):e0168424. doi: 10.1371/journal.pone.0168424 (PMC5240978; doi:10.1371/journal.pone.0168424)
Supplement: S3 Table — Limb-onset ALS patients showed white matter atrophy in motor and adjacent areas. In contrast, the bulbar-onset group showed more widespread involvement of white matter tracts, including motor and extra-motor regions. (DOCX) [file pone.0168424.s005.docx]

| Supporting 3 Table. Relative decrease in white matter volume of bulbar- and limb-onset ALS patients (compared to controls) | | | | | | | | | |
| --- | --- | --- | --- | --- | --- | --- | --- | --- | --- |
|  |  |  | | MNI-space | | | | |  |
| Type | AAL regions | Side | | Clusters | x | y | z | T-value | P-value |
| Limb | Caudate nucleus | | Right | 3800 | 12 | -8 | 20 | 5.65 | 0.000 |
|  | Paracentral lobule | | Right | 4769 | 6 | -28 | 74 | 6.60 | 0.000 |
|  | Paracentral lobule | | Right | 4769 | 4 | -38 | 74 | 5.69 | 0.000 |
|  | Supplementary motor area | | Left | 4769 | -2 | -14 | 64 | 5.43 | 0.000 |
|  | Superior temporal gyrus | | Left | 442 | -56 | -6 | 4 | 4.44 | 0.006 |
|  | Insula | | Left | 442 | -42 | -4 | 8 | 4.40 | 0.006 |
|  | Heschl's gyrus | | Left | 442 | -38 | -20 | 12 | 4.14 | 0.006 |
|  | Insula | | Right | 151 | 40 | -2 | 8 | 3.94 | 0.084 |
|  | Insula | | Right | 151 | 38 | 12 | 6 | 3.68 | 0.084 |
|  | Insula | | Right | 151 | 42 | -10 | 4 | 3.53 | 0.084 |
|  | [Lobule IX of vermis](http://neuro.imm.dtu.dk/w/index.php?title=Category:Lobule_IX_of_vermis&action=edit&redlink=1) | |  | 134 | 0 | -50 | -32 | 3.87 | 0.101 |
|  |  | |  |  |  |  |  |  |  |
| Bulbar | Cerebellum | | Left | 1080 | -4 | -62 | -34 | 4.36 | 0.000 |
|  | Postcentral gyrus | | left | 981 | -64 | -4 | 18 | 4.61 | 0.001 |
|  | Postcentral gyrus | | left | 981 | -40 | -10 | 48 | 4.48 | 0.001 |
|  | Middle frontal gyrus | | left | 981 | -36 | 14 | 52 | 4.48 | 0.001 |
|  | Supplementary motor area | | Right | 454 | 12 | 12 | 58 | 5.08 | 0.013 |
|  | Superior frontal gyrus | | right | 454 | 20 | 16 | 62 | 4.71 | 0.013 |
|  | Precentral gyrus | | Right | 289 | 24 | -18 | 64 | 3.93 | 0.039 |
|  | Parahippocampal gyrus | | right | 287 | 18 | -10 | -22 | 4.72 | 0.040 |
|  | Parahippocampal gyrus | | right | 287 | 24 | -2 | -32 | 3.50 | 0.040 |
|  | Fusiform gyrus | | right | 287 | 30 | -2 | -44 | 3.54 | 0.040 |
|  | Middle frontal gyrus | | Right | 261 | 42 | 2 | 54 | 4.20 | 0.048 |
|  | Middle frontal gyrus | | Right | 261 | 52 | -2 | 48 | 3.79 | 0.048 |
|  | Middle frontal gyrus | | Right | 207 | 42 | 46 | 16 | 3.78 | 0.075 |
|  | Medial frontal gyrus (orbitalis) | | right | 176 | 6 | 56 | -8 | 4.55 | 0.097 |
|  | Medial frontal gyrus (superior) | | right | 176 | 10 | 62 | 10 | 3.67 | 0.097 |
|  | Supplementary motor area | | Left | 232 | -14 | 6 | 62 | 4.42 | 0.061 |
|  | Postcentral gyrus | | Left | 163 | -26 | -36 | 50 | 3.62 | 0.109 |
|  |  | |  |  |  |  |  |  |  |
